# Factors That Influence the Use of Electronic Diaries in Health Care: Scoping Review

**DOI:** 10.2196/19536

**Published:** 2021-06-01

**Authors:** Naomi E M Daniëls, Laura M J Hochstenbach, Catherine van Zelst, Marloes A van Bokhoven, Philippe A E G Delespaul, Anna J H M Beurskens

**Affiliations:** 1 Department of Psychiatry and Neuropsychology Faculty of Health, Medicine and Life Sciences Maastricht University Maastricht Netherlands; 2 Department of Family Medicine Faculty of Health, Medicine and Life Sciences Maastricht University Maastricht Netherlands; 3 Research Centre for Remote Health Care Faculty of Health Care Zuyd University of Applied Sciences Heerlen Netherlands; 4 Mondriaan Mental Health Trust Heerlen/Maastricht Netherlands

**Keywords:** compliance, delivery of health care, diary, ecological momentary assessment, intention, motivation, scoping review

## Abstract

**Background:**

A large number of people suffer from psychosocial or physical problems. Adequate strategies to alleviate needs are scarce or lacking. Symptom variation can offer insights into personal profiles of coping and resilience (detailed functional analyses). Hence, diaries are used to report mood and behavior occurring in daily life. To reduce inaccuracies, biases, and noncompliance with paper diaries, a shift to electronic diaries has occurred. Although these diaries are increasingly used in health care, information is lacking about what determines their use.

**Objective:**

The aim of this study was to map the existing empirical knowledge and gaps concerning factors that influence the use of electronic diaries, defined as repeated recording of psychosocial or physical data lasting at least one week using a smartphone or a computer, in health care.

**Methods:**

A scoping review of the literature published between January 2000 and December 2018 was conducted using queries in PubMed and PsycInfo databases. English or Dutch publications based on empirical data about factors that influence the use of electronic diaries for psychosocial or physical purposes in health care were included. Both databases were screened, and findings were summarized using a directed content analysis organized by the Consolidated Framework for Implementation Research (CFIR).

**Results:**

Out of 3170 articles, 22 studies were selected for qualitative synthesis. Eleven themes were determined in the CFIR categories of intervention, user characteristics, and process. No information was found for the CFIR categories inner (eg, organizational resources, innovation climate) and outer (eg, external policies and incentives, pressure from competitors) settings. Reminders, attractive designs, tailored and clear data visualizations (intervention), smartphone experience, and intrinsic motivation to change behavior (user characteristics) could influence the use of electronic diaries. During the implementation process, attention should be paid to both theoretical and practical training.

**Conclusions:**

Design aspects, user characteristics, and training and instructions determine the use of electronic diaries in health care. It is remarkable that there were no empirical data about factors related to embedding electronic diaries in daily clinical practice. More research is needed to better understand influencing factors for optimal electronic diary use.

## Introduction

Health care professionals are insufficiently aware of symptom variability and contextual fluctuations; therefore, their interventions are based on incomplete information [1-5]. Patients are asked to recall their mood, thoughts, behavior, and experiences over the past weeks or even months. Recalling information from memory, though, is known to be incomplete and inaccurate [6,7]. To minimize inaccuracies and biases, prospective diaries are used to collect patients’ mood, thoughts, behavior, and experiences in the relevant context close to the time of occurrence [8]. Because these health-related strategies often require management of vulnerabilities, long-term patient engagement is important. However, patients experience that it is difficult to be engaged in the use of diaries for long periods of time. Compliance is often poor, and adequate reports on contextual variation are lacking [8]. Paper diaries are remarkably completed in the parking lot before meeting the clinician [9]. In one-third of the days, paper diaries contain entries while the log booklets were not opened [8,10].

To overcome noncompliance with paper diaries, researchers and clinicians have shifted from paper to electronic diaries. Both paper and electronic diaries can be used in research to observe individuals in their context, gather data about sensitive topics, or to actively engage individuals in monitoring and reflecting on behaviors, their underlying mechanisms, and processes. Furthermore, these diaries can be implemented in intervention studies, clinical trials, and routine care [11,12]. Electronic diaries are, however, more reliable and logistically easier to implement [13,14]. They allow individuals to monitor in daily life with little retrospection and reduced obtrusiveness. Electronic diaries are signal-contingent and often record response-time information, which improves reliability [15-18]. Nonetheless, electronic diaries also have disadvantages. Development and maintenance are costly [12]. Technical problems occur, and not all patients are acquainted with smartphones and require instructions and coaching [15]. Furthermore, research on compliance is ambiguous. For instance, the percentage of completed diary entries with electronic diaries ranges from less than 50% to 99% [18-20]. High participant motivation is related to accurate data collection and less faked compliance [13].

Previous research states that various factors are related to the use of electronic diaries, such as the design (ie, ease of use, entertainment value), the social context (ie, satisfaction and connection with others), and the user’s characteristics (ie, education and self-efficacy) [21-23]. However, no complete overview is available concerning empirical data about the factors related to the use of these tools. Therefore, the main aim of this paper was to map the existing empirical knowledge about the factors that influence the use of electronic diaries in health care. Electronic diaries in health care were defined as repeated individual psychosocial or physical data collection using measurement tools on a smartphone (applications) or on a computer (website), including among others, experience sampling, ambulatory assessment, and ecological momentary assessment. In addition, use was defined as the repeated recording of information in electronic diaries by patients or healthy individuals for at least one week, including adherence, compliance, and engagement. The cut-off point was determined based on the expected recall bias and necessary data for comprehensive functional diagnostics.

## Methods

In order to map existing knowledge concerning the topic of interest and to identify any gaps, this scoping review was based on the methodological framework proposed by Arksey and O’Malley [24]. This framework includes 5 specific steps: identify the research question, identify relevant studies, select relevant studies, chart the data, and summarize and report the results. The selection of relevant studies was not based on methodological quality, but on relevance.

### Identify the Research Question

The research question of this scoping review was based on prior research and the expertise of the research team. It is summarized as: “What is the current empirical knowledge regarding factors that influence the use of electronic diaries in health care?”

### Identify and Select Relevant Studies

A structured literature search was conducted using the PubMed and PsycInfo databases to search for articles published between 2000 and 2018. The search was limited to human adults and articles published in Dutch or English. Both free-text search terms and MESH headings were used. The search strategy included 2 different concepts: “continued use” and “electronic diaries.” The search string used is depicted in Textbox 1. In addition to the database search, reference lists of relevant studies were screened manually for further relevant papers. This is a valuable step (snowball method) to identify articles that have been missed in the database search because electronic databases may be incomplete and they can vary in coverage, indexing, and depth of information [24]. Moreover, 2 experts in the field were contacted to identify key authors or key publications on the topic of interest.

**Search string.**

1. Use: “compliance (MeSH) OR intention (MeSH) OR motivation (MeSH) OR ‘continued usage’ OR use OR continuance OR adherence OR engagement”;

AND

2. Electronic diaries: “momentary (MeSH: ecological momentary assessment) OR ‘real time data’ (MeSH) OR e-diaries OR electronic diar* OR structured diar* OR computer diar* OR ‘experience sampling’ OR ambulatory assessment OR electronic assessment* OR electronic interview* OR self-monitoring”

Limits:

- Publication date: 2000-2018
- Humans: adult
- Language: English, Dutch

Two researchers (NEMD, LMJH) reviewed the retrieved studies using a 3-step screening process: titles, abstracts, and full articles. The screening process of a scoping review is not linear but rather iterative, which required the researchers to engage with each step in a reflexive way and repeat steps to ensure that the literature was covered in an extensive way. If the relevance of a study was unclear from the title, the abstract was ordered, and if the relevance of a study was unclear from the abstract, the full article was ordered. As a check on the 3-step screening process, we read the full texts of a random sample of 50 titles and 50 abstracts. In only 4 articles, we found information in the results or the discussion related to our scope. Relevant studies with the following criteria were included: (1) using electronic diaries for psychosocial or physical data, (2) describing factors that influence the use of electronic diaries, and (3) a focus on health care. No methodological criteria were applied, and articles based on empirical data were included. Studies were excluded when the definitions of electronic diaries or use in the article did not match with the ones used in this manuscript (ie, the data collection method: single moment data collection or passive self-monitoring using sensors, activity trackers, or biomarkers). Studies that used a combination of active and passive monitoring were not excluded. Moreover, studies were excluded when the article did not include factors that influence the use of electronic diaries as the outcome (ie, the study aim: experiences with disease management, epidemiology, health technology assessment, prediction models, outcome and effect studies, and the study design [reviews, secondary analysis, protocols]). Studies in which disease management were based on or complemented with self-reporting and studies about technology acceptance were not excluded. Furthermore, we excluded studies with a target population other than adults. For children and adolescents, we expect that different factors influence the use of electronic diaries specifically and interventions in general as parents, for instance, need to give their permission. At each step, the articles were categorized as relevant, irrelevant, and dubious according to the aforementioned exclusion criteria. Differences were discussed until consensus was reached. When no consensus was reached or questions remained, a third researcher (CvZ) was consulted.

### Chart the Data

The data were charted using Excel spreadsheets and included study details (author, title, database, journal, year of publication, study location [published and conducted], study population and sample size, study aims, design, and setting), intervention characteristics (aim, content, and duration of the electronic diary), and key findings (factors that influence the use). These factors were organized according to the Consolidated Framework for Implementation Research (CFIR) [25]. This framework consists of 5 categories (ie, intervention characteristics, outer setting, inner setting, individual characteristics, and process) related to sustainable implementation. The intervention characteristics category includes, among others, the complexity of the electronic diary or the ability to test the electronic diary on a small scale. The outer setting category is comprised of the economic, political, and social context of the organization. The inner setting category includes, among others, the internal architecture of the organization and the innovation climate. The individual characteristics category is comprised of, among others, the individual’s knowledge, beliefs, and self-efficacy regarding the intervention or the implementation process. The process category includes activities (planning, engaging, executing, reflecting, and evaluating) related to the implementation process.

### Summarize and Report the Results

Content analysis was done independently by 2 reviewers (NEMD, LMJH) based on the 5 categories of the CFIR [25]: (1) intervention, (2) outer setting, (3) inner setting, (4) individual characteristics, and (5) process. Directed content analysis, using inductive reasoning, was used to validate or conceptually extend the framework [26]. The themes were based on our previous work [27] and emerged from the data. After coding, the researchers compared their codes until consensus was reached. They identified key themes into which the results could be divided.

## Results

**Figure 1:**
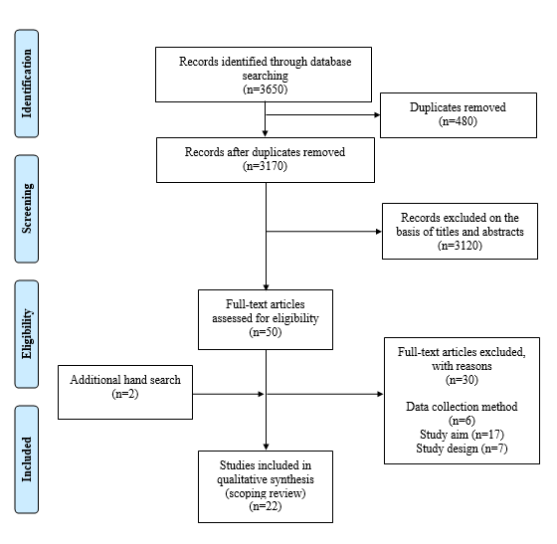
Scoping review flow diagram.

The database search resulted in 3650 hits (Figure 1). After removing duplicates and reviewing 3170 titles, 273 abstracts were screened, of which 50 full texts were evaluated. In total, 20 articles were included based on the predefined eligibility criteria. Two articles were included from the additional hand search, which resulted in 22 articles in total for qualitative synthesis. The publication patterns are summarized in Multimedia Appendix 1.

### Electronic Diary and Study Characteristics

More detailed information about the content of the 22 selected studies with empirical data on factors that influence the use of electronic diaries in health care can be found in Table 1. Electronic diaries were used either to monitor one’s own behavior in order to get insight into underlying patterns or mechanisms (monitoring: 12/22, 55%) or to actively achieve change (intervention: 10/22, 45%). They mainly focused on measuring lifestyle behaviors (14/22, 64%) and constructs such as pain or mood. Participants completed these electronic diaries via palmtop (3/22, 14%), smartphone (14/22, 64%), or (tablet) computer (5/22, 22%). The assessment frequency ranged from 12 times a day, an example of the experience sampling method or ecological momentary assessment (EMA), to weekly, and the duration of the data collection varied from 2 weeks to 2.5 years.

**Table 1:** Electronic diary (e-diary) and study characteristics.

First author, year, country	e-Diary characteristics				Study characteristics		
	Purpose of use^a^ (device)	Constructs measured	Frequency of use and duration	Study aims		Design and data collection	Sample: target population, number of participants, sex, age (years)
Aaron, 2004 [28], US	Intervention: cognitive behavioral therapy-based pain management training (palmtop)	Pain intensity, pain-related activity interference, jaw use limitations, mood, perceived stress	3 times a day for 8 weeks	Self-reported reasons for missing electronic diary interviews (EMA^b^)		Quantitative: secondary analysis of existing RCT^c^ data (CBT^d^-based pain management training or self-care manual condition)	Patients with TMD^e^ (n=62), 16% male (n=10), mean age 38.6 (SD 11.6)
Litcher-Kelly, 2007 [29], US	Monitoring: self-monitoring diaries (palmtop)	Mood, stress, pain, medication use	12 times a day for 3 weeks	Feasibility of an electronic diary		Quantitative: intervention study with continuous log data	Patients with inflammatory bowel disease (n=16), 25% male (n=4), mean age 46.0 (SD 13.6)
Welch, 2007 [30], US	Monitoring: self-monitoring diaries (palmtop)	Food and fluid intake	3 times a day for 12 weeks	Feasibility of electronic self-monitoring diaries		Quantitative: pilot study with surveys	Patients on hemodialysis (n=3), 67% male (n=2), mean age 54
Stevens, 2008 [31], US	Intervention: IT^f^ weight loss program (computer)	Weight, food records, exercise minutes	Weekly for a 2.5-year follow-up	First year utilization and development process of an IT weight loss program		Quantitative: RCT with 3 groups (no-further treatment, control condition, or active maintenance weight loss intervention)	Adults with a BMI of 25-45 kg/m^2^ who were taking medication for hypertension or hyperlipidemia (n=348), 37% male (n=128), mean age 56
Webber, 2010 [32], US	Monitoring: internet behavioral weight loss program (computer)	Daily caloric intake, daily exercise, weight	At least weekly for 16 weeks	Motivation and adherence to self-monitoring and weight loss		Quantitative: secondary analysis of existing RCT data (did or did not achieve 5% weight loss)	Adult women with a BMI of 25-40 kg/m^2^ (n=66), mean age 50.1 (SD 9.9)
Ahtinen, 2013 [33], Finland	Intervention: Oiva, a mobile mental wellness training application (smartphone)	Reflections and notes on exercises	Daily for a month	Use, acceptance, and usefulness of Oiva		Mixed methods: feasibility study with surveys, app log data and interviews	Individuals interested in stress management (n=15), 40% male (n=6), working age
Ben-Zeev, 2013 [34], US	Intervention: FOCUS, a mobile illness self-management system (smartphone)	Medication adherence, mood regulation, sleep, social functioning, coping with persistent auditory hallucinations	Daily	Development of FOCUS		Mixed methods: usability study with surveys and think-aloud procedure	Patients with schizophrenia or schizoaffective disorder (n=12), 67% male (n=8), mean age 45
Ma, 2013 [35], US	Intervention: eHealth weight loss intervention (computer)	Weight, physical activity	12 weeks, no app use criteria	Acceptance and use of an eHealth weight management intervention		Quantitative: secondary analysis of existing RCT data (coach-led or self-directed group)	Overweight or obese adults with prediabetes and/or metabolic syndrome (n=133), 53% male (n=70), mean age 53.5 (SD 10.5)
Tatara, 2013 [36], Norway	Monitoring: Few Touch, a mobile self-management application (smartphone)	Nutritional habits	1 year, no app use criteria	Factors associated with use of Few Touch, a mobile self-management application		Mixed methods: longitudinal intervention trial with surveys, interviews, and focus groups	Individuals with type 2 diabetes mellitus (n=12), 33% male (n=4), mean age 55.1 (SD 9.6)
Tang, 2015 [37], UK	Monitoring: publicly available free applications MyFitness Pal, Livestrong, Calorie Count, SparkPeople (smartphone)	Not specified	3 weeks, no app use criteria	Understanding of users’ experiences with weight loss or weight control apps		Qualitative: semistructured interviews	Young adults having experience with or interest in using an eHealth weight loss maintenance app (n=19), 54% male (n=10), age range 19-33
Triantafyllidis, 2015 [38], UK	Monitoring: SUPPORT-HF, a remote health monitoring and nonpharmacological, self-monitoring system (tablet computer)	Physiological measurements (blood pressure, weight, oxygen saturation), heart failure symptoms, quality of life	5 days a week for 1 year	Development of SUPPORT-HF		Mixed methods: iterative refinement approach informed by action research	Patients with heart failure (n=26), 65% male (n=17), mean age 72 (SD 15)
Anderson, 2016 [39], Australia	Monitoring: applications about chronic conditions (sleep disorders, migraine, menstrual irregularities, chronic depression, arthritis and Behçet’s disease; smartphone)	Ranging from symptom monitoring or management apps to fitness apps	Ranging from several weeks to 2 years	Consumers’ experiences with mobile health apps		Qualitative: individual semistructured interviews	Healthy individuals reporting the recent use of any commercially available health/fitness app with capacity for self-monitoring and data input (n=22), 32% male (n=7), age range 18-55
Batink, 2016 [40], The Netherlands	Intervention: ACT-DL, a mobile acceptance and commitment therapy in daily life training (smartphone)	Sleep quality, appraisal of the day, affect (positive and negative feelings), cognition, context (activity, company and whereabouts)	10 times a day for 3 days each week, for 4 weeks	Feasibility, acceptability, and effectiveness of ACT-DL (EMI^g^)		Mixed methods: intervention study with 2 groups (experimental intervention or outpatient treatment)	Patients with a mental health disorder such as anxiety, mood, somatoform, or substance disorders: experimental intervention (n=49), 35% male (n=17), mean age 45.7 (SD 10.0); healthy individuals (n=112), 55% male (n=62), mean age 47.5 (SD 12.4)
Jiang, 2016 [41], US	Monitoring: Pocket Personal Assistant for Training Health (Pocket PATH), a health self-monitoring application (smartphone)	Spirometry, temperature, blood pressure, pulse, symptoms, weight	12 months posttransplantation, no app use criteria	Acceptance and use of Pocket PATH		Quantitative: cross-sectional correlational design with secondary analysis of existing RCT data	Lung transplantation recipients transferred to the acute cardiothoracic unit (n=96), 51% male (n=49), mean age 57 (SD 14)
Naughton, 2016 [42], UK	Intervention: Q-sense, a smoking cessation mobile phone application (smartphone)	Smoking behavior, psychological context, situational context	1 month before until 2 weeks after a preset quit date	Feasibility of Q-sense (EMI)		Mixed methods: an explanatory sequential mixed methods design with app log data and semistructured interviews	Adult smokers willing to set a quit date in the period between 1 week and 1 month after inclusion (n=15), 53% male (n=8), age range 18-45
Timmerman, 2016 [43], The Netherlands	Monitoring: telehealth care application with a symptom monitoring module and web-based exercise module (smartphone and computer)	Pain, fatigue, dyspnea	3 days a week during 2 weeks presurgery, the first month postsurgery, and 2 weeks prior to the doctor consultation at 3 and 6 months postsurgery	Development and usability of a multimodal ICT^h^-supported rehabilitation program for lung cancer		Qualitative: user-centered design with interviews and focus groups	Patients with NSCLC^i^ (n=10), 30% male (n=3), mean age 62 (SD 11)
Burke, 2017 [44], US	Intervention: standard behavioral intervention for weight (smartphone)	Not specified	5 times a day for 12 months	Lessons learned from development and implementation of an EMA study, focusing on the methods and logistics of conducting an EMA study and including strategies to ensure adequate adherence to EMA prompts		Qualitative: single-group, observational design	Former participants of laboratory weight loss studies (n=133), 9% male (n=12), mean age 51.09 (SD 10.10)
Crane, 2017 [45], UK	Monitoring: DrinkLess, an application (smartphone)	Consequences of alcohol consumption, mood, productivity, clarity, sleep quality	Daily, at least 2 weeks, no app use criteria	Usability of DrinkLess		Qualitative: usability studies with think-aloud procedure and semistructured interviews	Healthy individuals (n=12 for both studies), 50% male (n=6), mean age 42 (first study) and 40 (second study)
Freyne, 2017 [46], Australia	Intervention: PMRP^j^, a behavioral-based mobile weight management program and application (smartphone)	Meal diary for previous day, current weight, dietary intake, update food diary	3 times a day for an intervention period of 12 weeks, followed by another 12-week period	Role of push notifications in persuading users to engage with self-monitoring tasks		Quantitative: intervention study with app log data	Overweight adults (BMI >25 kg/m^2^; n=75), 27% male (n=20), mean age 48.6
Kreyenbuhl, 2018 [47], US	Intervention: MedActive, an application (smartphone)	Medication adherence, positive psychotic symptoms, medication side effects	Daily for 2 weeks	Acceptability and feasibility of MedActive (EMA)		Quantitative: user-centered design with surveys	Patients with schizophrenia spectrum disorder taking ≥1 oral antipsychotic medications (n=7), 100% male (n=7), mean age 47.6 (SD 10.4)
Liu, 2018, US [48]	Monitoring: LoseIt, a physical activity and diet tracking application (smartphone)	Food intake	At least 3 days a week for 2 weeks	Effectiveness of LoseIt		Quantitative: randomized trial with 2 groups (goal setting reminders or generic reminders) with pre- and posttests	College students (n=50), 38% male (n=19), mean age 21 (SD 1.8)
Tomko, 2018 [49], US	Monitoring: REDCap, ambulatory assessment software (computer)	Smoking, substance use, medication adherence	3 times daily for 8 weeks	Feasibility of ambulatory assessment (here applied in smoking cessation) for research purposes (EMA)		Quantitative: feasibility study within a double-blind RCT with 2 groups (N-acetylcysteine or placebo)	Adult smokers (n=36), 50% male (n=18), mean age 41.1 (SD 12.7)

^a^The purpose of use category is based on the authors’ interpretation of the described goal of the electronic diary.

^b^EMA: ecological momentary assessment.

^c^RCT: randomized controlled trial.

^d^CBT: cognitive behavioral therapy.

^e^TMD: temporomandibular disorder.

^f^IT: information technology.

^g^EMI: ecological momentary intervention.

^h^ICT: information communication technology.

^i^NSCLC: non-small cell lung cancer.

^j^PMRP: partial meal replacement program.

The factors that influence the use of electronic diaries in health care were not the primary aim in all included studies. These factors were mentioned as part of a larger study, such as a randomized controlled trial or an intervention study. Studies focused on usability in half of the articles (10/22, 45%), followed by feasibility and effectiveness (7/22, 32%) and development (5/22, 23%). The design of these studies was quantitative (11/22, 50%), mixed (6/22, 27%), or qualitative (5/22, 23%). The number of participants ranged from 3 to 348, with a mean age of 49 years. Of these, 37.0% (493/1341) were male. The majority of the studies included patients with physical symptoms (12/22, 55%), whereas healthy individuals (7/22, 32%) and patients with mental health symptoms (3/22, 13%) were less often described.

### Factors That Influence the Use of Electronic Diaries

**Figure 2:**
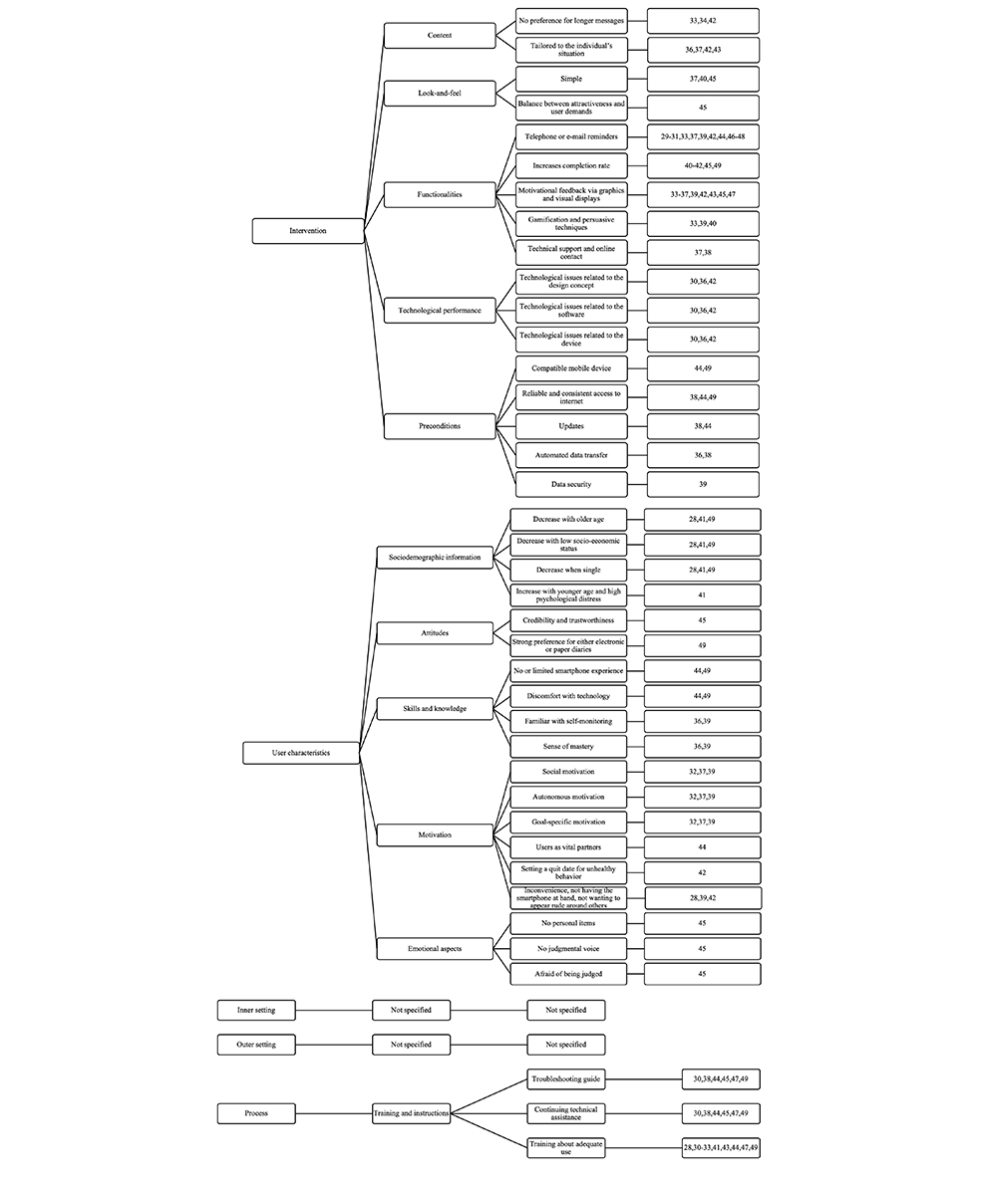
Visual representation of the factors that influence the use of electronic diaries in health care.

The CFIR [25] was used to perform the qualitative thematic analysis of the factors that influence the use of electronic diaries in health care. The results of this qualitative thematic analysis were organized along 3 CFIR categories: intervention [29-31,33-49], user characteristics [28,32,36,37,39,41,42,44,45,49], and process [30-33,38,41,43-45,47,49]. No results were found for the 2 other CFIR categories: inner setting and outer setting. Figure 2 gives an overview of these categories, themes, and subthemes.

#### Intervention

The first category describes the key attributes of an electronic diary device, a smartphone application, or a web-based module. Five themes specify the intervention.

The first theme, “content,” refers to the information in an electronic diary. Smartphone applications and web-based modules consisted of several content types like EMA, reminders, and reward messages [33,40,45,47-49]. This content supports communication between the patient and the health care professional. Long messages are considered too time-consuming to read, and users would therefore skip screens [33,34,42]. Furthermore, users may prefer both cartoons or videos and text [40-42,45,49]. Moreover, diary questions should be tailored to the individual’s situation [36,37,42,43]. Users are inconclusive about the scope of the constructs measured; some may prefer an exclusive focus on one topic, whereas others may find that too limited [37,47].

The second theme, “look and feel,” refers to the configuration or layout of an electronic diary. The user interface should be both simple and attractive [37,40,45]. However, a balance between attractiveness and user demands is required. Users may prefer a visually appealing user interface with minimal demands on them [45].

The third theme, “functionalities,” refers to the activities that a user can perform within an application, ranging from procedures for recording and uploading data to customization of the user interface. Telephone or email reminders, either programmable or automated, notify the user to complete a questionnaire, which increases the completion rate [29-31,33,37,39,42,44,46-48]. Furthermore, manually entering several indicators per day increases participant burden [40-42,45,49]. Moreover, users want to receive motivational feedback about their results via clear graphics and visual displays [33-37,39,42,43,45,47]. Gamification and persuasive techniques can be used to provide motivational feedback to increase completion rates [33,39,40]. Additionally, Tang et al [37] and Triantafyllidis et al [38] identified that technical support and online contact with, for example, a health care professional increase the use of an electronic diary.

The fourth theme, “technological performance,” refers to the technological issues that users encounter while using an electronic diary. Users can experience technological issues related to the design concept (eg, navigation problems), the software, or the device (eg, battery attrition). These errors reduce the usability of an electronic diary [30,36,42].

The fifth theme, “preconditions,” refers to the conditions that must be fulfilled before a smartphone application or a web-based module can function properly. Burke et al [44] and Tomko et al [49] suggested that users are provided with a compatible mobile device (with sufficient memory, processing speed, and a functioning camera) to overcome the barrier of installing additional hardware or software on the user’s device. Moreover, Burke et al [44], Tomko et al [49], and Triantafyllidis et al [38] stated that users need reliable and consistent access to the internet while using the tool. Furthermore, they suggested checking for operating system and other smartphone updates that potentially interfere with the smartphone application of interest [38,44]. The electronic diary should be updated continuously; hence, bandwidth limitations should be taken into account, especially for web-based modules [31]. Automated data transfer to the background server or another device must be seamless for the individual to be able to use the device with minimal effort [36,38]. Depending on the type of data, users highly value data security. They are especially concerned that data would not be shared with health insurers [39].

#### User Characteristics

The second category describes the characteristics of the individuals who use the electronic diary, in this case, healthy individuals and patients with physical or psychosocial problems. Five themes specify the user characteristics.

The first theme, “sociodemographic information,” refers to the characteristics of a population such as gender, age, and marital status. The use of an electronic diary decreases when individuals are older, have a low socioeconomic status, or are unmarried, separated, divorced, or widowed [28,41,49], whereas an increase in the use of these tools is seen when individuals experience high psychological distress [41].

The second theme, “attitudes,” refers to the way a user feels and behaves with regard to an electronic diary. Crane et al [45] concluded that users’ positive attitudes towards smartphone applications or web-based modules are based on credibility and trustworthiness of the information. Moreover, Tomko et al [49] stated that users may have strong preferences for either electronic or paper diaries.

The third theme, “skills and knowledge,” refers to the information that a user has about electronic diaries and the ability to use these tools. Users with no or limited smartphone experience and who experience discomfort with technology will not use electronic diaries adequately. Extra staff is required to train these users [44,49]. Additionally, users who become familiar with self-monitoring or get a sense of mastery over their problems will lose their motivation and consequently stop or reduce their app use [36,39].

The fourth theme, “motivation,” refers to the needs, desires, and drives of the individual to use an electronic diary. Naughton et al [42], Anderson et al [39], and Aaron et al [28] stated that missing data are not caused by low motivation, but by discomfort, not having the smartphone at hand, or not wanting to appear rude around others. Social motivation, autonomous motivation, and goal-specific motivation increase the adherence to using electronic diaries [32,37,39]. Furthermore, making users vital partners in the development of an electronic diary keeps them motivated to use these devices [44]. In case of unhealthy behaviors, setting a quit date boosts users’ commitment [42].

The fifth theme, “emotional aspects,” refers to the feelings that are induced by using an electronic diary. When diary questions are too personal or judgmental, users are less likely to engage with a smartphone application or a web-based module [45]. Furthermore, they want to keep their data private because they are afraid of being judged [45]. However, in the study by Aaron et al [28], emotional aspects were the least mentioned reasons for missing a questionnaire, although Crane et al [45] found that users feel guilty when diaries are missed.

#### Process

The third category describes the activities related to the implementation process. One theme specifies the process.

The theme, “training and instructions,” refers to how users are guided and instructed to adequately use an electronic diary. Training (eg, face-to-face group kick-off presentation, training session to familiarize with the tool and troubleshoot issues) could result in higher use of these tools [28,30-33,41,43,44,47,49]. Furthermore, users may prefer a troubleshooting guide with step-by-step instructions or continuing technical assistance in case of technological issues from the staff or development team [30,38,44,45,47,49].

## Discussion

### Principal Findings

This scoping review maps the existing knowledge and gaps concerning factors that influence the use of electronic diaries in health care. Due to technological developments in the last decades, electronic diaries have become increasingly available and popular in research and routine clinical practice. This increased interest is also visible in the large number of articles published between 2000 and 2018. However, only a small number of these articles focused on factors that influence the use of electronic diaries. Additionally, an even smaller number of the selected articles focused on implementing these tools in daily clinical practice.

In this scoping review, 22 articles were selected based on the predefined eligibility criteria. For the categories of intervention, user characteristics, and process of the CFIR [25], 11 themes were identified, whereas no empirical data were found for the 2 other CFIR categories: inner setting and outer setting. The use of an electronic diary is facilitated when it is a visually appealing tool with various content types, including reminders, clear in-app data visualizations tailored to the individual, and minimal user demands to increase the user’s engagement. A compatible mobile device with reliable internet access and automated data transfer supports adequate use of an electronic diary. Additionally, the user needs to have smartphone experience, intrinsic motivation, and a clear rationale to monitor one’s own behavior. Finally, both theoretical training and practical training are recommended to foster the implementation process. However, the required content and procedures of such training were not described in the included studies.

Based on these results and considering relevant implementation and adoption models, 2 findings attract attention. First, it is remarkable that there were only empirical data about the influence of the characteristics of the electronic diary, the individual, and the implementation process, whereas the CFIR and other implementation frameworks also emphasize the importance of factors related to the organization in which the care is provided or the organizational culture (inner setting) and the competition or the pressure from external partners and the regulations or legislation concerning electronic diaries in clinical practice (outer setting) [25]. Recent research on the implementation of patient-reported outcome measures also highlights the importance of investing sufficient time and resources to support health care professionals [50-54].

Second, the scope of the implementation framework CFIR, used in this review, appears to be wider than adoption models that are traditionally used to evaluate user engagement and continued use of information systems and mobile technologies, like the Technology Acceptance Model [55-59]. The adoption models limit the scope to characteristics of the electronic diary and the individual user, whereas the CFIR also takes into account the process of implementation in daily clinical practice. In this review, the importance of training and instructions was revealed. The importance of hands-on instructions (individual coaching on the job sessions to familiarize with the use of experience-sampling technology in daily clinical practice, using real-world examples) as well as the ability to contact a help desk in case of practical and technological issues was underlined in our previous study as well [27]. Also, regarding the characteristics of the electronic diary, the adoption models have a smaller focus. They only highlight the running software as a contributing factor, while this scoping review identified that the information about and the layout of these diaries, as well as the technological issues and preconditions, also influence their use [55-59]. However, when considering the characteristics of the individual user, this scoping review revealed personal characteristics such as age, along with attitudes, emotions, and behaviors, while adoption models also focus on social influence and self-efficacy as contributing factors [55-59].

Implementation literature emphasizes that attention should be paid to the range of influencing factors to achieve a successful implementation in daily clinical practice [25,50-54]. Consequently, sustainable use of electronic diaries requires that health care organizations or professionals not only direct attention towards software, hardware, and the target population of the tool but also to the economic and political organizational context, the innovation climate in the organization, and the embedding of the tool in routine clinical practice.

### Strengths and Limitations

Several limitations have to be kept in mind while interpreting the results of this scoping review. The structured literature search was based on a combination of key words defined by preliminary literature exploration and expert consultation. Despite a broad search approach, it is still possible that articles were missed since the research topic was often not the primary aim of the included studies. This possibly resulted in selection bias. However, the additional hand search minimized this potential shortcoming. It is also worth noting that most of the articles were excluded based on title screening. This can be seen as a limitation, but we think this approach is justifiable in our sensitive search. We performed an iterative screening process that required the researchers to engage in a reflexive way and repeat steps to ensure that the literature was covered in an extensive way. When the relevance of the study was not clear from the title, the abstract was always read. But it is still possible that we missed some articles. Moreover, as an extra check on the 3-step screening process, we read the full texts of a random sample of 50 titles and 50 abstracts. In only 4 articles, we found information in the results or the discussion related to our scope. Furthermore, as the aim of this scoping review was to map the existing empirical knowledge and identify any gaps about factors that influence the use of electronic diaries in health care, no study quality assessment was performed. Moreover, a scoping review does not endeavor to give a summary of the existing literature or compare results (in contrast to a systematic review of, for example, randomized controlled trials on efficacy). Therefore, we did not intend to draw firm conclusions regarding useful and effective features of electronic diaries based on quantified outcomes. We provide, to our knowledge, a first overview of the factors that influence the use of electronic diaries in health care. Future research with longitudinal or mixed methods study designs should focus on the causal relationships between the influencing factors and the use of electronic diaries in health care in order to get a deeper understanding of the causality. Also, a quite diverse sample of studies was included. However, we are convinced that we have achieved the scope of interest of this scoping review. We looked in more detail at similarities and differences in the results of the included studies, based on the purpose of use (monitoring versus intervention), target population (healthy individuals versus patients), setting, study aims, and design (feasibility versus usability versus development). However, we concluded that this synthesis cannot be performed based on the results of the information found in this scoping review. More research is needed in this field. Additionally, the structured literature search was restricted to peer-reviewed databases and so, empirical research. Book chapters and grey literature were not included, which means that additional empirical data can be lacking. This scoping review has several methodological strengths as well. First, a systematic approach was used based on the methodological framework by Arksey and O’Malley [24]. The interprofessional nature of the research team extended the scope of this review, and the consultation of 2 experts in the field validated the search terms. Furthermore, the 3-step screening process was consistently performed by 2 researchers. Second, the thematic analysis organized according to an implementation research perspective led to a synthesis contributing to future understanding of the implementation of electronic diaries in health care.

### Conclusion

This scoping review demonstrates that the use of electronic diaries may be influenced by characteristics of the electronic diary, the individual user, and the implementation process. However, the number of empirical studies on the topic was limited. Studies that take into account the setting in which to implement the diaries, such as the organizational context, the implementation climate, and available organizational resources, were lacking. Future research should focus on these factors and on the causal relationships between the different factors to investigate the continued use of these innovative tools.

## Appendix

Multimedia Appendix 1 Table S1. Patterns of publications.

## Abbreviations

CFIR: Consolidated Framework for Implementation Research
EMA: ecological momentary assessment
