# Supplementary material for: Factors That Influence the Use of Electronic Diaries in Health Care: Scoping Review
Source: JMIR Mhealth Uhealth. 2021 Jun 1;9(6):e19536. doi: 10.2196/19536 (PMC8207255; doi:10.2196/19536)
Supplement: Multimedia Appendix 1 [file mhealth_v9i6e19536_app1.docx]

Table S1. Patterns of publications

| Main aspect | Distribution | N (%) |
| --- | --- | --- |
|  |  |  |
| Database | PubMed | 14 (64%) |
|  | PsycInfo | 8 (36%) |
| Publication date | 2000-2009 | 4 (18%) |
|  | 2010-2014 | 5 (23%) |
|  | 2015-2018 | 13 (59%) |
| Countries | United States of America | 12 (54%) |
|  | United Kingdom | 4 (18%) |
|  | The Netherlands | 2 (9%) |
|  | Norway | 1 (5%) |
|  | Australia | 2 (9%) |
|  | Finland | 1 (5%) |
